# *An. gambiae* gSG6-P1 evaluation as a proxy for human-vector contact in the Americas: a pilot study

**DOI:** 10.1186/s13071-015-1160-3

**Published:** 2015-10-13

**Authors:** Berlin Londono-Renteria, Papa M. Drame, Thomas Weitzel, Reinaldo Rosas, Crystal Gripping, Jenny C. Cardenas, Marcela Alvares, Dawn M Wesson, Anne Poinsignon, Franck Remoue, Tonya M. Colpitts

**Affiliations:** Department of Pathology, Microbiology and Immunology, University of South Carolina, Columbia, SC USA; Laboratory of Parasitic Diseases, National Institutes of Health, NIAID, Bethesda, MD USA; Laboratorio Clínico/Programa Medicina del Viajero, Clínica Alemana, Universidad del Desarrollo, Santiago, Chile; Hospital Militar, Santiago, Chile; Department of Tropical Medicine, Tulane University, New Orleans, LA USA; Hospital Los Patios, Los Patios, Norte de Santander Colombia; Hospital Emiro Quintero Canizales, Ocana, Norte de Santander Colombia; Institut de Recherche pour le Développement-IRD, Bouaké, Côte d’Ivoire; Department of Pathology, Microbiology and Immunology, University of South Carolina School of Medicine, 6439 Garners Ferry Rd, Bldg 2 Rm C3, Columbia, SC 29209 USA

## Abstract

**Background:**

During blood meal, the female mosquito injects saliva able to elicit an immune response in the vertebrate. This immune response has been proven to reflect the intensity of exposure to mosquito bites and risk of infection for vector transmitted pathogens such as malaria. The peptide gSG6-P1 of *An. gambiae* saliva has been demonstrated to be antigenic and highly specific to *Anopheles* as a genus. However, the applicability of gSG6-P1 to measure exposure to different *Anopheles* species endemic in the Americas has yet to be evaluated. The purpose of this pilot study was to test whether human participants living in American countries present antibodies able to recognize the gSG6-P1, and whether these antibodies are useful as a proxy for mosquito bite exposure and malaria risk.

**Methods:**

We tested human serum samples from Colombia, Chile, and the United States for the presence of IgG antibodies against gSG6-P1 by ELISA. Antibody concentrations were expressed as delta optical density (ΔOD) of each sera tested in duplicates. The difference in the antibody concentrations between groups was tested using the nonparametric Mann Whitney test (independent groups) and the nonparametric Wilcoxon matched-pairs signed rank test (dependent groups). All differences were considered significant with a *P* < 0.05.

**Results:**

We found that the concentration of gSG6-P1 antibodies was significantly correlated with malaria infection status and mosquito bite exposure history. People with clinical malaria presented significantly higher concentrations of IgG anti-gSG6-P1 antibodies than healthy controls. Additionally, a significant raise in antibody concentrations was observed in subjects returning from malaria endemic areas.

**Conclusion:**

Our data shows that gSG6-P1 is a suitable candidate for the evaluation of exposure to *Anopheles* mosquito bites, risk of malaria transmission, and effectiveness of protection measures against mosquito bites in the Americas.

## Background

Globally, an estimated 3.3 billion people are at risk of being infected with malaria [1, 2]. Although the majority of cases and deaths occur in Africa, approximately 170 million people in the Americas live at risk of malaria infection, with 74 % of cases caused by *Plasmodium vivax* and 25 % by *P. falciparum* [3, 4]. Malaria is transmitted to humans when the infective sporozoites, located in salivary glands, are injected into the human skin through the bite of a female *Anopheles* mosquito. These sporozoites are submerged in mosquito saliva [5] that is used by the mosquito to facilitate blood uptake. Mosquito saliva contains physiologically active components able to counteract blood coagulation, active complement, and the response to hinder the bite injury. Interestingly, some of these salivary components can also elicit and modify immune responses in the vertebrate host and can therefore modify the outcome of vector-transmitted diseases [6, 7].

The gold standard to measure the intensity of malaria transmission is the entomological inoculation rate (EIR), which is defined as the number of infectious *Anopheles* bites per person in a given time [8]. EIR measurement is highly dependent on the density of human-biting *Anopheles* [9]. The latter is estimated by trapping methods such as human-landing catches (HLCs) of adult mosquitoes. However, the technique of HLC poses ethical concerns as the human “bait” could be exposed to transmission of malaria and other mosquito-borne diseases. In addition, this trapping technique is only applicable to human adults. It is difficult to extrapolate HLC results to children or to pregnant woman that are the most vulnerable to malaria [10]. As an alternative, catching traps such as the CDC light trap, CDC light trap associated with CO_2_ or the Mbitrap have been developed [11]. However, studies have shown that these alternative methods have several limitations, like preferentially capturing mosquitoes with higher sporozoite rate and consequently overestimating EIR [12] and can not replace HLC [13]. Therefore, new tools able to evaluate vector–host contact as well as the changes of this contact over time are needed to monitor both population and individual exposure and disease risk.

Previous works have shown that the immune response against mosquito salivary proteins, specially IgG antibodies, can reflect the intensity of exposure to mosquito bites as well as the risk of infection for vector-borne pathogens [14–16]. The serological evaluation of this immune response and its association with the exposure to malaria vectors is receiving increasing attention, especially regarding the major malaria vectors in Africa and India, *An. gambiae sensu lato* and *An. stephensi*, respectively. Transcriptome analysis of the salivary glands of *An. gambiae*, identified a protein named *An. gambiae* salivary gland protein 6 (gSG6), a member of the SG protein family known to be exclusively expressed in the adult *Anopheles* female [17]. Most of the SG proteins seem to be very well conserved among Old World species, *An. gambiae*, *An. arabiensis*, and *An. funestus*. However, clustal alignment of gSG6 showed similarities with the New World *Anopheles* species, *An. quadrimaculatus* and *An. freeborni* [18, 19]. The peptide gSG6 is restricted to the *Anopheles* genus based on substantial previous research, which has also demonstrated that specific IgG response to gSG6-P1 could serve as a biomarker for exposure to malaria vectors [2, 17, 19–25]. Moreover, the use of the gSG6-P1 salivary peptide has enabled the collection of relevant data on the efficacy of long lasting insecticide treated nets (LLIN) over time [26] and to compare the effectiveness of individual protection tools such as spray bombs and mosquito coils to LLINs [27].

The development of accurate and sensitive tools to identify variations in vector exposure and malaria risk is important to assess the effectiveness of control efforts. So far, the relevance of antibody response against gSG6-P1 has only been evaluated in subjects exposed to bites of *Anopheles* species in the Old World. The purpose of this study was to evaluate whether gSG6-P1 is a suitable biomarker for exposure to bites of American species of *Anopheles*, serving as an indicator of the efficacy of individual/collective malaria vector control tools and a proxy for *Plasmodium* transmission risk in the New World.

## Methods

### Ethics statement

Universidad de Pamplona, Los Patios Hospital in Norte de Santander-Colombia, approved this study. Additionally, an IRB approval from University of North Carolina at Chapel Hill was granted for the previously described double-blind RCT examining effects of permethrin-treated uniforms on the incidence of tick bites in North Carolina outdoor workers. The Military Hospital and the Chilean Army Health Command in Santiago, Chile, granted the approval for the inclusion of Chilean military personnel. Written consent was obtained from each volunteer.

### Study participants

#### Chile

A sample of Chilean soldiers participating for a period of 6 months in the United Nations Stabilization Mission in Haiti (MINUSTAH) in 2011 were included in the present study (*n* = 45) (Table 1). They were part of a surveillance project of the Department of Preventive Medicine of the Chilean Military to evaluate the risk of vector-borne disease for peace troops working in Haiti. Samples were obtained from troops that were stationed in Port-au-Prince and responsible for road and bridge building. Daily activities included exposure to swamps, rivers, and flooded roads, as well as diurnal and nocturnal patrolling and convoy security duties in different rural sites. All soldiers received training to avoid mosquito exposure before departure. In Haiti, measures against vector-borne infections included personal use of skin repellent containing 34 % DEET (Ultrathon™, 3M, St. Paul, MN, USA), impregnation of occupational clothes with a permethrin-based repellent, weekly oral dose of 300 mg of chloroquine (base), sleeping in container-type facilities with air-conditioning, and indoor residual spraying and space spraying (adulticiding) with permethrin derivate every 15 days. During off-duty hours troops were authorized to wear sport and casual clothing. Almost all soldiers spent their 15-days UN leave at beach resorts in the Dominican Republic. The main malaria vector in both Haiti and the Dominican Republic is *An. albimanus* [28, 29]. Serum samples were collected before and after returning from Haiti and kept at −20 °C until processing.

#### Colombia

In Colombia, the highest reported altitude for *Anopheles* species is 2000 m above the sea level (m.a.s.l.) [30]. For this study, we included 26 participants (Colombia population 1), aged 18 to 21 years, living in a malaria free area located at 2300 m.a.s.l. in Pamplona City, but traveling for at least 30 days to endemic areas in the Caribbean Coast of Colombia (States of Guajira, Cordoba, Sucre, Magdalena and Santander), where the main malaria vectors are *An. albimanus*, *An. darlingi*, and *An. punctimacula* [31]. These participants were included in a follow-up study to test reactivity to vector salivary proteins before and after traveling to endemic areas. Participants were bled before and within a week after returning to Pamplona.

A second group from Colombia (Colombia population 2) was formed by febrile participants (*n* = 42) seeking malaria diagnosis and medical services at the Local Hospital of Los Patios and Ocana in the Norte de Santander department, which is a known low malaria endemic area, and at the San Francisco de Asis Hospital in Quibdo in the Choco department, considered the second highest endemic area for malaria in the country. Serum samples were collected and kept at −20 °C until processing.

**Table 1:** Sample description

Country	Sample description	Sample groups	*n*	Species potential exposure
Chile	Chilean soldiers serving in Haiti	Before Deployment	45	*An. albimanus*
		Return	45	
Colombia	Healthy subjects	Before traveling	26	
		After traveling	26	*An. albimanus*
				*An. darlingi*
				*An. punctimacula*
	Febrile patients	Malaria (+)	7	
		Malaria (−)	35	
USA	Park Rangers	Before protective clothing	42	*An. punctipennis*
				*An. quadrimaculatus*
		After protective clothing	42	

#### United States

We selected 42 North Carolina Parks and Forestry rangers participating in a randomized controlled trial to test the efficacy of long-lasting permethrin-impregnated (LLPI) clothing. Serum samples were collected on time 1 (before implementation of protective clothing) and time 2 (after implementation of protective clothing) from participants of the control group (*n* = 24) and the test group (*n* = 18). Aliquots of the samples were kept at −20 °C until testing.

### Malaria testing by PCR

Total DNA was isolated from human samples using the gDNA Blood Kit (Life technologies, Carlsbad, CA, USA) according to manufacturer’s instructions. Plasmodium-specific PCR was performed using a multiplex PCR for the small sub-unit (SSU) rRNA gene described elsewhere [32]. DNA from in vitro culture of the Haiti I/CDC strain of *P. falciparum* and a blood sample from a thick-smear *P. vivax* positive patient were used as positive controls.

### Antibody detection by ELISA

The ELISA conditions followed in this study were published elsewhere [26]. Briefly, 96-well ELISA plates were coated with 100 μL/well of 0.5 μg/ml of gSG6-P1 in coating solution (Kirkegaard & Perry Laboratories, Inc. Gaithersburg, MD) serum samples were incubated at 37 °C for 2 h in a 1/100 dilution. After washing, plates were incubated with 100 μL/well of horseradish peroxidase (HRP)-conjugated goat anti-human IgG (1:1000) antibodies at 37 °C for 1.5 h. Later, each well received 100 μL/well of tetra-methyl-benzidine (TMB) (Gene-Script, Piscataway, NJ) and 100 μL/well solution of 1 M phosphoric acid to stop the reaction after 5 min incubation with the TMB. Absorbance was measured at 450 nm.

### Statistical analysis

Antibody concentrations were expressed as delta optical density (ΔOD) calculated for each sample subtracting the mean OD value of the negative control and blank wells from the mean OD value of the duplicates for each sample. Positive control ΔOD values from all plates were recorded and averaged. This average was then divided by individual plate positive control ΔOD values to obtain a “calculation factor” for each plate. To correct for plate-to-plate variations, we then multiplied each sample ΔOD value by their respective plate calculation factor to obtain normalized ΔOD’s. The difference in the antibody concentrations between two independent groups was tested using the Mann Whitney *U* test while the difference between two dependent groups was calculated using Wilcoxon matched-pairs rank test. To determine risk of disease by Odd Ratios (OR), we divided the study sample into two groups with high and low IgG anti-gSG6-P1 antibody levels. As a threshold the median ΔOD of 1.3075 was used. Significant differences were established at p values higher lower 0.05. All statistical testing was performed with Prism version 6.0 (Graph Pad Software Inc.) and STATA™ version 10.1 (Stata Corporation).

## Results and discussion

### Anti-gSG6-P1 IgG as biomarker of exposure to “New World” *Anopheles* mosquito bites

**Fig. 1:**
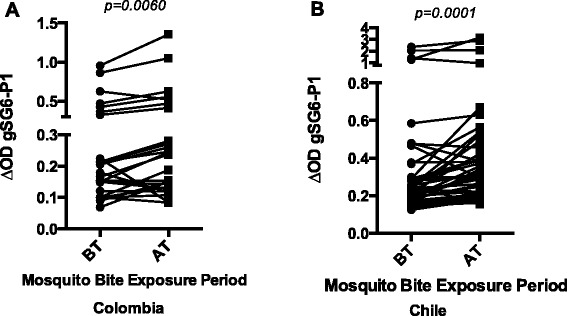
IgG antibody levels against gSG6-P1 before (BT) and after traveling (AT) to a malaria endemic area. **a** Healthy civilian form Colombia living in a malaria-free area, traveling to malaria endemic places during vacation. **b** Military personnel from Chile deployed in Haiti in humanitarian mission. *p* value denote significance by the Wilcoxon matched-pairs signed rank test

Estimating mosquito-human contact rate in endemic areas for vector transmitted diseases like malaria, is an important tool to measure the risk of transmission of such diseases. Since saliva form arthropods is injected at the time of transmission, efforts has been directed into discover the salivary proteins able to elicit an immune response that correspond to the level of bites a person has received in a determined period of time. Recently, the protein gSG6 form *An. gambiae*, has been reported as a useful marker for the degree of exposure to mosquito bites in malaria endemic areas of Africa. Research shows that the gSG6 protein presents little sequence variation within species of the *An. gambiae* complex (i.e. *An. melas*, *An. quadriannulatu*, *An. arabiensis*). However, sequence comparison of the gSG6 with more distant related species like *An. stephensi* (Asia) and *An. freeborni* (America) shows a 67 to 71 % sequence identity [17]. There is not available information on the sequence similarity between gSG6 and the South or Central American Anopheles species An. albimanus, An. darlingi or An. nuneztovari). Nevertheless, salivary transcriptome analysis of the Nyssorhynchus subgenus did not reveal SG6 presence in the salivary content of these species [33, 34]. These reports suggested that a possible reason for this divergence is an independent evolutionary event of the gSG6 protein among anopheline subgenera although they also state that more data was necessary to confirm that hypothesis. In spite of the few available information on the American species homology with gSG6 protein [17, 35], several reports showed the gSG6 protein to be specific to the genus *Anopheles*. Consequently, we wanted to evaluate whether participants exposed to bites of American endemic *Anopheles* species develop antibodies specific to gSG6-P1, a salivary antigen validated as a biomarker of exposure to *An. gambiae s. l*. and *An. funestus* (main malaria vectors in Africa) bites. To do this, we used the gSG6-P1 peptide to assess exposure to *Anopheles* bites in healthy participants from Colombia living in a malaria-free area before and after travel to endemic malaria regions (Fig. 1a). In this group, we noticed a significant increase of individual levels of anti-gSG6-P1 IgG after traveling (AT) to malaria areas in comparison to those before traveling (BT) (*P* = 0.006). A similar increase in specific gSG6-P1 IgG levels was observed in Chilean soldiers after a 6-months stay in malaria endemic areas in Haiti and the Dominican Republic (*P* < 0.0001; Fig. 1b). These results suggest that IgG response to gSG6-P1 is closely associated with the degree of exposure to the bites of *An. albimanus*, *An. darlingi* or *An. punctimacula*, the malaria vectors described in these endemic areas in Latin America.

These results implicate that gSG6-P1 peptide, although based on the Old World *An. gambiae s. s*. mosquito, can be used to evaluate the level of exposure to bites of New World (Americas) species of *Anopheles* malaria vectors. Previous studies conducted in Senegal, Angola, and Benin have described a correlation between the level of gSG6-P1 IgG antibodies and the intensity of human exposure to *Anopheles* bites [21, 22, 26]. Cross-response to gSG6-P1 peptide bases on the fact that gSG6 is a well conserved polypeptide presenting homologues in both the Old and New World *Anopheles* species [36]. In conclusion, gSG6 polypeptide as well as its gSG6-P1 peptide is suitable as a “bite” biomarker of Old World as well as New World species of *Anopheles* malaria vectors.

### IgG specific to gSG6-P1 as a proxy of malaria transmission risk

**Fig. 2:**
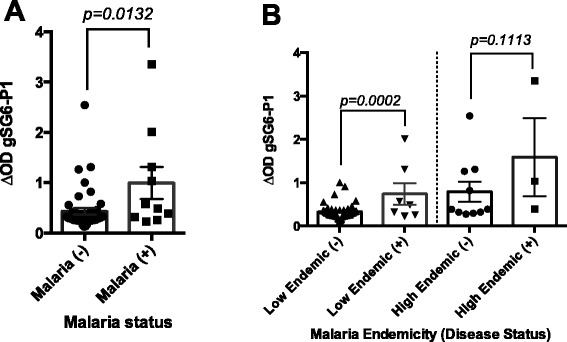
IgG anti-gSG6-P1 antibody levels according to malaria status in Colombian participants. The results are presented by malaria status determine by PCR as malaria positive (+) or negative (−) (**a**), and by malaria endemicity classified as “low” or “high” endemic area (**b**). *p* value denote significance by the by the Mann Whitney *U* test

Anti-gSG6-P1 IgG levels were assessed in malaria negative (−) and malaria positive (+) groups of participants residing in malaria endemic areas of Colombia where *An. albimanus*, *An. darlingi*, and *An. punctimacula* are the main vectors. Our results demonstrated that the median level of specific IgG antibodies against gSG6-P1 was significantly higher in the malaria infected group compared to the uninfected group (*P* = 0.0132) (Fig. 2a). Serum samples in Colombia were collected in two different states: 1) Choco with an annual parasitic incidence (API) of 49 cases/1000 habitants, which in 2010 was classified as a high malaria transmission region [37] and 2) Norte de Santander, which is classified as low transmission area (API: 1.3 cases/1000). Interestingly, the difference in median anti-gSG6-P1 IgG levels between malaria infected and uninfected participants was only significant in participants living in areas of low malaria endemicity (*P* = 0.0002). In the high transmission area, no significant difference in anti-gSG6-P1 levels was observed (*P* = 0.1113) (Fig. 2b).

**Fig. 3:**
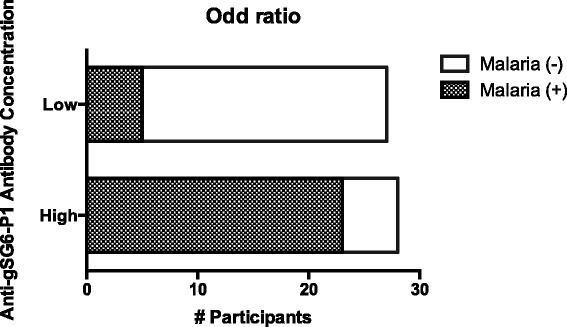
Risk evaluation of malaria using IgG antibody levels against gSG6-P1 peptide. Fisher Exact Test *p* = 0.0001

We also tested the hypothesis that IgG-gSG6-P1 antibody concentration serves as a proxy for the risk of acquiring malaria. For that, we divided our study subjects into two categories: “low antibody levels” (with a ΔOD value <0.774) and “high antibody levels” (with a ΔOD value ≥0.775). Participants with “high” levels of IgG anti-gSG6-P1 antibodies had a 20.24 times higher risk for acquiring malaria (*P* = 0.0001) than people with lower levels of those antibodies (Fig. 3) suggesting that more mosquito bites elicit higher anti-gSG6-P1 antibodies and that higher antibody levels are also associated with a higher possibility of being exposed to a bite from an Plasmodium-infected mosquito. This suggests that the level of gSG6-P1 specific IgG could be used as biomarker for the risk of malaria infection. Our data confirm previous studies conducted in different parts of the Old World, in particular a recent study in a low to moderate malaria transmission area of Senegal, which reported an association between IgG response to gSG6-P1 and the status *P. falciparum* infection of children, even during the dry season [22]. Furthermore, this study showed different levels of anti-gSG6- IgG P1 antibodies in asymptomatic *Plasmodium* carriers compared to clinical malaria cases. In Kenya, an association between the prevalence of *P. falciparum* and the levels of anti-gSG6-P1 IgG was also described [38]. Another study in Burkina-Faso revealed a positive correlation between malaria incidence and IgG response against the gSG6 recombinant protein [39]. All together, the anti-salivary gSG6-P1 peptide IgG response, previously demonstrated to be a biomarker of exposure to *Anopheles* bites in numerous contexts and in this study, could also be a useful indicator of malaria exposure and infection. This could also be useful to estimate the malaria risk for non-indigenous populations taking chemoprophylaxis such as the Chilean soldiers of our study, which helps to adapt protection strategies for these populations. In a high transmission situation in Colombia, we found no significant differences in anti-gSG6-P1 levels of malaria (+) and malaria (−) groups suggesting that the biomarker might have limitation in this setting. Although the sample size of this study group was small, it suggested that in hyperendemic malaria areas, residents might be highly exposed to infectious and none-infectious bites. They thus present high levels of anti-gSG6-P1 antibodies, which do not necessarily reflect the risk of malaria transmission. Therefore, for such situations there is a need for more specific biomarkers.

It is important to clarify that these findings are applicable for malaria transmission settings in the absence of antimalarial drugs as prophylactic treatments. In such cases, individuals will be still exposed to vector bites with a reduced risk of presenting symptoms and evaluation of the gSG6 antibody levels will be suitable marker for human-vector contact.

In the African settings where previous studies with gSG6-P1 took place, entomological records were made to evaluate the abundance of *Anopheles* species present in these areas. So, it was known that the majority of the *Anopheles* species present in these areas were potential vectors of malaria. In Colombia, between 70 and 90 % of the mosquitoes captured during entomological surveillance belong to genus *An. albimanus*, *An. darlingi* or *An. nuneztovari* considered the main vectors of malaria in the country [40, 41] while *An. albimanus* is the most abundant *Anopheline* specie in the Hispaniola Island [42, 43]. However, If same studies will be undertaken in area where only half of the *Anopheles* are malaria vectors, we might observe the same non significance between infected and non-infected individuals, as in hyperendemic transmission area, probably because people would receive as many uninfected that infected bites and no association between the level of IgG to gSG6-P1 peptide and the risk of transmission would be observed.

### Human IgG response to gSG6-P1 for evaluating effectiveness of individual protection against mosquito bites

**Fig. 4:**
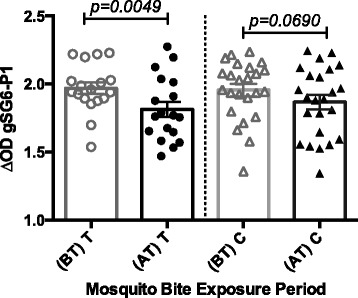
Level of anti-gSG6-P1 IgG antibodies in park rangers using anti-mosquito bite protective clothing (T) and controls (C) on year 1 (basal antibody levels–before intervention (BT) and follow up levels on year 2 (AT). *p* value denote significance by the Wilcoxon matched-pairs signed rank test

A major application for an immunologically based vector-human contact biomarker is the assessment of the efficacy of vector control methods. For instance, Drame et al. proved that the use of insecticide-treated bednets and spray bombs drastically reduced the IgG levels specific to gSG6-P1 peptide, independently of age or transmission season [22, 27]. Other studies also suggest that the IgG response against gSG6-P1 is a reliable alternative to accurately assess the effectiveness of malaria control methods and surveillance programs in areas of low malaria risk [44, 45]. In this study, we evaluated and compared the IgG-gSG6-P1 levels of US park rangers using LLPI clothing during working hours and control participants wearing untreated clothes before and after the implementation of the strategy. At baseline, both groups presented similar levels of IgG specific to gSG6-P1 peptide. One year after the implementation of LLPI clothing, we observed a significant decrease in the concentration of IgG-gSG6-P1 antibodies in the treatment group (*P* = 0.0049), whereas no changes were observed in the controls (Fig. 4).

*Anopheles punctipennis* is the most abundant *Anopheles* species in North Carolina (US), mostly biting outdoors after dusk. However, it will attack humans also during daytime in dense woodlands or close to their daylight resting places, where the park rangers might be performing their work activities. Monitoring the efficacy of individual use of anti-malaria vector tools is very challenging. Previous data suggested that the gSG6-P1 salivary biomarker can be used to assess and monitor the effectiveness of individual or collective protection tools/strategies against *Anopheles* mosquito bites [22, 26, 27]. Our study confirmed this by showing that the level of *Anopheles* bites was significantly reduced in those rangers wearing LLPI clothes. We therefore believe that gSG6-P1 salivary biomarker is a useful marker to assess and monitor the effectiveness of protection measures also against New World *Anopheles* bites. This raises the importance of personal protection to vector bites in the control of malaria. This is in accordance with previous data showing significant reduction of specific IgG levels, human-*Anopheles* contact and bites in association with the use of spray bombs [26]. However all personal protection tools do not seem to reduce the level of mosquito bites as pointed by the same authors who described a none-significant effect of the use of mosquito coils or electric fans/air conditioning. In addition, by comparing the efficacy of individual protection tools such as spray bombs and mosquito coils to bed-nets, the same authors indicate that the decreases in *Anopheles* aggressiveness has essentially been due to the use of bed-nets, meaning that these individual protection tools can be complementary but cannot replace bed-nets in the fight against malaria.

In the case of the Chilean troops in Haiti, air conditioning facilities and bednets were used for all the troops at base. Additionally, they use of military uniforms, left neck and wrists exposed so although most of the body is protected these areas can still reached by mosquitoes during the night work. These observations suggest that the use of these uniforms, reinforced by chloroquine treatment, reduced the risk of presenting malaria in the presence of higher mosquito bite exposure (There has been only one case of malaria in 10 years of mission in Haiti), and that gSG6-P1 is a useful marker to show that in the event of increased exposure to mosquito bites subjects were protected against infection.

## Conclusion

The synthetic peptide gSG6-P1 is useful as a proxy indicator of human-vector contact with American *Anopheles* species. Further investigation is needed to compare the immune response against the specific mosquito species vectors of malaria in America with the response elicited against gSG6-P1 in areas with different malaria endemicity. It is also a useful tool for the evaluation vector control interventions and devices designed to decrease mosquito bite and disease transmission in human populations.

## References

[1] Nilsson SK, Childs LM, Buckee C, Marti M (2015). Targeting human transmission biology for malaria elimination. PLoS Pathog.

[2] WHO Malaria Report 2014. WWMR. 2014.

[3] Guerra CA, Gikandi PW, Tatem AJ, Noor AM, Smith DL, Hay SI, Snow RW (2008). The limits and intensity of Plasmodium falciparum transmission: implications for malaria control and elimination worldwide. PLoS Med.

[4] Quintero JP, Siqueira AM, Tobon A, Blair S, Moreno A, Arevalo-Herrera M, Lacerda MV, Valencia SH (2011). Malaria-related anaemia: a Latin American perspective. Mem Inst Oswaldo Cruz.

[5] Barabutis N, Siejka A, Schally AV, Block NL, Cai R, Varga JL (2010). Activation of mitogen-activated protein kinases by a splice variant of GHRH receptor. J Mol Endocrinol.

[6] Schneider BS, Higgs S (2008). The enhancement of arbovirus transmission and disease by mosquito saliva is associated with modulation of the host immune response. Trans R Soc Trop Med Hyg.

[7] Schneider BS, Mathieu C, Peronet R, Mecheri S (2011). Anopheles stephensi saliva enhances progression of cerebral malaria in a murine model. Vector Borne Zoonotic Dis.

[8] Rajamanonmani R, Nkenfou C, Clancy P, Yau YH, Shochat SG, Sukupolvi-Petty S, Schul W, Diamond MS, Vasudevan SG, Lescar J (2009). On a mouse monoclonal antibody that neutralizes all four dengue virus serotypes. J Gen Virol.

[9] Birley MH, Charlewood JD (1987). Sporozoite rate and malaria prevalence. Parasitol Today.

[10] Duffy PE, Fried M (2005). Malaria in the pregnant woman. Curr Top Microbiol Immunol.

[11] Kline DL (2006). Traps and trapping techniques for adult mosquito control. J Am Mosq Control Assoc.

[12] Mboera LE (2005). Sampling techniques for adult Afrotropical malaria vectors and their reliability in the estimation of entomological inoculation rate. Tanzan Health Res Bull.

[13] Hiwat H, Andriessen R, Rijk M, Koenraadt CJ, Takken W (2011). Carbon dioxide baited trap catches do not correlate with human landing collections of Anopheles aquasalis in Suriname. Mem Inst Oswaldo Cruz.

[14] Margos G, Navarette S, Butcher G, Davies A, Willers C, Sinden RE, Lachmann PJ (2001). Interaction between host complement and mosquito-midgut-stage Plasmodium berghei. Infect Immun.

[15] Londono-Renteria BL, Eisele TP, Keating J, James MA, Wesson DM (2010). Antibody response against Anopheles albimanus (Diptera: Culicidae) salivary protein as a measure of mosquito bite exposure in Haiti. J Med Entomol.

[16] Londono-Renteria B, Cardenas JC, Cardenas LD, Christofferson RC, Chisenhall DM, Wesson DM, McCracken MK, Carvajal D, Mores CN (2013). Use of anti-Aedes aegypti salivary extract antibody concentration to correlate risk of vector exposure and dengue transmission risk in Colombia. PLoS One.

[17] Lombardo F, Ronca R, Rizzo C, Mestres-Simon M, Lanfrancotti A, Curra C, Fiorentino G, Bourgouin C, Ribeiro JM, Petrarca V (2009). The Anopheles gambiae salivary protein gSG6: an anopheline-specific protein with a blood-feeding role. Insect Biochem Mol Biol.

[18] Ndille EE, Dubot-Peres A, Doucoure S, Mouchet F, Cornelie S, Sidavong B, Fournet F, Remoue F (2014). Human IgG antibody response to Aedes aegypti Nterm-34 kDa salivary peptide as an indicator to identify areas at high risk for dengue transmission: a retrospective study in urban settings of Vientiane city, Lao PDR. Trop Med Int Health.

[19] Ribeiro JM, Mans BJ, Arca B (2010). An insight into the sialome of blood-feeding Nematocera. Insect Biochem Mol Biol.

[20] Poinsignon A, Cornelie S, Mestres-Simon M, Lanfrancotti A, Rossignol M, Boulanger D, Cisse B, Sokhna C, Arca B, Simondon F (2008). Novel peptide marker corresponding to salivary protein gSG6 potentially identifies exposure to Anopheles bites. PLoS One.

[21] Poinsignon A, Cornelie S, Ba F, Boulanger D, Sow C, Rossignol M, Sokhna C, Cisse B, Simondon F, Remoue F (2009). Human IgG response to a salivary peptide, gSG6-P1, as a new immuno-epidemiological tool for evaluating low-level exposure to Anopheles bites. Malar J.

[22] Sagna AB, Sarr JB, Gaayeb L, Drame PM, Ndiath MO, Senghor S, Sow CS, Poinsignon A, Seck M, Hermann E (2013). gSG6-P1 salivary biomarker discriminates micro-geographical heterogeneity of human exposure to Anopheles bites in low and seasonal malaria areas. Parasit Vectors.

[23] Drame PM, Machault V, Diallo A, Cornelie S, Poinsignon A, Lalou R, Sembene M, Dos Santos S, Rogier C, Pages F (2012). IgG responses to the gSG6-P1 salivary peptide for evaluating human exposure to Anopheles bites in urban areas of Dakar region, Senegal. Malar J.

[24] Rizzo C, Ronca R, Fiorentino G, Verra F, Mangano V, Poinsignon A, Sirima SB, Nebie I, Lombardo F, Remoue F (2011). Humoral response to the Anopheles gambiae salivary protein gSG6: a serological indicator of exposure to Afrotropical malaria vectors. PLoS One.

[25] Orlandi-Pradines E, Almeras L, Denis de Senneville L, Barbe S, Remoue F, Villard C, Cornelie S, Penhoat K, Pascual A, Bourgouin C (2007). Antibody response against saliva antigens of Anopheles gambiae and Aedes aegypti in travellers in tropical Africa. Microbes Infect.

[26] Drame PM, Poinsignon A, Besnard P, Cornelie S, Le Mire J, Toto JC, Foumane V, Dos-Santos MA, Sembene M, Fortes F (2010). Human antibody responses to the Anopheles salivary gSG6-P1 peptide: a novel tool for evaluating the efficacy of ITNs in malaria vector control. PLoS One.

[27] Drame PM, Diallo A, Poinsignon A, Boussari O, Dos Santos S, Machault V, Lalou R, Cornelie S, LeHesran JY, Remoue F (2013). Evaluation of the effectiveness of malaria vector control measures in urban settings of Dakar by a specific anopheles salivary biomarker. PLoS One.

[28] Hobbs JH, Sexton JD, St Jean Y, Jacques JR (1986). The biting and resting behavior of Anopheles albimanus in northern Haiti. J Am Mosq Control Assoc.

[29] Mekuria Y, Williams DC, Tidwell MA, Santana TA (1990). Studies of the susceptibility of Anopheles albimanus and Anopheles vestitipennis from Dajabon, Dominican Republic, to insecticides. J Am Mosq Control Assoc.

[30] Molina AM (2008). Sistemas de Informacion Geografica para el Analisis de la Distribucion Espacial de la Malaria en Colombia. Revista EIA.

[31] Víctor Alberto Olano HLB, Sáenz R, Quiñones ML, Molina JA (2001). Mapas preliminares de la distribución de especies de Anopheles vectores de malaria en Colombia. Biomedica.

[32] Padley D, Moody AH, Chiodini PL, Saldanha J (2003). Use of a rapid, single-round, multiplex PCR to detect malarial parasites and identify the species present. Ann Trop Med Parasitol.

[33] Calvo E, Andersen J, Francischetti IM, deL Capurro M, deBianchi AG, James AA, Ribeiro JM, Marinotti O (2004). The transcriptome of adult female Anopheles darlingi salivary glands. Insect Mol Biol.

[34] Calvo E, Pham VM, Marinotti O, Andersen JF, Ribeiro JM (2009). The salivary gland transcriptome of the neotropical malaria vector Anopheles darlingi reveals accelerated evolution of genes relevant to hematophagy. BMC Genomics.

[35] Rizzo C, Ronca R, Fiorentino G, Mangano VD, Sirima SB, Nebie I, Petrarca V, Modiano D, Arca B (2011). Wide cross-reactivity between Anopheles gambiae and Anopheles funestus SG6 salivary proteins supports exploitation of gSG6 as a marker of human exposure to major malaria vectors in tropical Africa. Malar J.

[36] Nagarajan S, Chesla S, Cobern L, Anderson P, Zhu C, Selvaraj P (1995). Ligand binding and phagocytosis by CD16 (Fc gamma receptor III) isoforms. Phagocytic signaling by associated zeta and gamma subunits in Chinese hamster ovary cells. J Biol Chem.

[37] Rodriguez JC, Uribe GA, Araujo RM, Narvaez PC, Valencia SH (2011). Epidemiology and control of malaria in Colombia. Mem Inst Oswaldo Cruz.

[38] Badu K, Siangla J, Larbi J, Lawson BW, Afrane Y, Ong’echa J, Remoue F, Zhou G, Githeko AK, Yan G (2012). Variation in exposure to Anopheles gambiae salivary gland peptide (gSG6-P1) across different malaria transmission settings in the western Kenya highlands. Malar J.

[39] Stone W, Bousema T, Jones S, Gesase S, Hashim R, Gosling R, Carneiro I, Chandramohan D, Theander T, Ronca R (2012). IgG responses to Anopheles gambiae salivary antigen gSG6 detect variation in exposure to malaria vectors and disease risk. PLoS One.

[40] Olano VA, Brochero H, Sáenz R, Quiñones M, Molina J (2001). Mapas preliminares de la distribución de especies de Anopheles vectores de malaria en Colombia. Biomedica.

[41] Olano V, Carrasquilla G, Mendez F (1997). [Transmission of urban malaria in Buenaventrua, Colombia: entomological features]. Pan Am J Public Health.

[42] Caillouet KA, Keating J, Eisele TP (2008). Characterization of aquatic mosquito habitat, natural enemies, and immature mosquitoes in the Artibonite Valley, Haiti. J Vector Ecol.

[43] Kiszewski A, Mellinger A, Spielman A, Malaney P, Sachs SE, Sachs J (2004). A global index representing the stability of malaria transmission. AmJTrop Med Hyg.

[44] Kuadkitkan A, Wikan N, Fongsaran C, Smith DR (2010). Identification and characterization of prohibitin as a receptor protein mediating DENV-2 entry into insect cells. Virology.

[45] Ribeiro JM, Arca B, Lombardo F, Calvo E, Phan VM, Chandra PK, Wikel SK (2007). An annotated catalogue of salivary gland transcripts in the adult female mosquito, Aedes aegypti. BMC Genomics.

